# The Recent Emergence of *Clostridium difficile* Infection in Romanian Hospitals is Associated with a High Prevalence of Polymerase Chain Reaction Ribotype 027

**DOI:** 10.4274/balkanmedj.2017.0400

**Published:** 2018-03-15

**Authors:** Gabriel Adrian Popescu, Roxana Serban, Adriana Pistol, Andreea Niculcea, Andreea Preda, Daniela Lemeni, Ioana Sabina Macovei, Daniela Tălăpan, Alexandru Rafila, Dragoş Florea

**Affiliations:** 1 Clinic of Infectious Diseases, National Institute for Infectious Diseases Matei Bals, Bucharest, Romania; 2 Department of Infectious Diseases, Carol Davila University School of Medicine, Bucharest, Romania; 3 National Centre for Communicable Surveillance and Control, National Institute for Public Health, Bucharest, Romania; 4 Clinic of Microbiology and Immunology, “Cantacuzino” National Institute for Research, Bucharest, Romania

**Keywords:** *Clostridium difficile*, epidemiology, ribotype 027, Romania

## Abstract

**Aims:**

To investigate the epidemiology of *Clostridium difficile* infection in Romanian hospitals.

**Methods:**

A survey was conducted at nine hospitals throughout Romania between November 2013 and February 2014.

**Results:**

The survey identified 393 patients with *Clostridium difficile* infection. The median age was 67 years (range: 2-94 years); 56% of patients were aged >65 years. The mean prevalence of *Clostridium difficile* infection was 5.2 cases per 10.000 patient-days. The highest prevalences were 24.9 and 20 per 10.000 patient-days in hospitals specializing in gastroenterology and infectious diseases, respectively. *Clostridium difficile* infections were health care-associated in 70.5% patients and community-acquired in 10.2%. The origin was not determined in 19.3%. *Clostridium difficile* infection was severe in 12.3% of patients, and the in-hospital all-cause mortality was 8.8%. Polymerase chain reaction ribotype 027 had the highest prevalence in all participating hospitals and represented 82.6% of the total ribotyped isolates. The minimum inhibitory concentration of moxifloxacin was >4 μg/mL for 59 of 80 tested isolates (73.8%). Of 59 isolates, 54 were highly resistant to moxifloxacin (minimum inhibitory concentration ≥32 μg/mL), and the majority were polymerase chain reaction ribotype 027 (p<0.0001).

**Conclusion:**

The ribotype 027 was the predominant cause of *Clostridium difficile* infections in Romania. In some specialized hospitals, the prevalence of *Clostridium difficile* infection was higher than the European mean prevalence, and this demonstrates the need for strict adherence to infection control programs.

The increasing incidence and severity of *Clostridium difficile* infections (CDIs) reported since 2001 in the United States and Canada (1,2) have been associated with the emergence of a hypervirulent ribotype 027 (2). The first European CDI outbreaks caused by this strain of *C. difficile* occurred in the United Kingdom and Ireland in 2004 (3) and were followed by spread of the ribotype from Western to Eastern Europe (4). A decrease in ribotype 027 infections in the United Kingdom has been reported following implementation of effective hospital infection control programs for CDI (5). The first confirmed cases of severe ribotype 027 CDI in Romania occurred in 2011. However, very few data regarding the *C. difficile* ribotypes in Romania are available, and most data have been obtained only from Bucharest and the surrounding area (6). The present epidemiological survey aimed to evaluate the spread of CDI in patients admitted to selected hospitals throughout Romania and determine the epidemiology (prevalence and the circulating ribotypes) and the frequency of occurrence of severe CDI cases.

## MATERIALS AND METHODS

**Figure 1:**
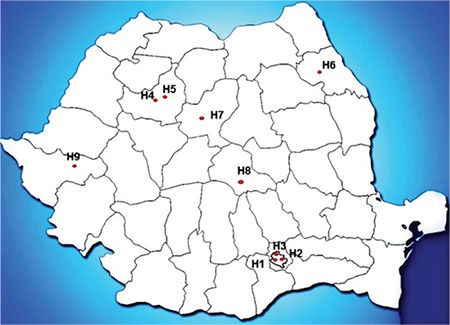
Participating hospitals in Bucharest (H1-H3), Cluj (H4-H5), Iasi (H6), Targu Mures (H7), Brasov (H8), and Timisoara (H9).

The study population included consecutive patients diagnosed with CDI and onset of symptoms between November 2013 and February 2014. The participating hospitals were located in the following six cities in different regions of Romania (Figure 1): Bucharest (H1-H3), Cluj (H4-H5), Iasi (H6), Targu Mures (H7), Brasov (H8) and Timisoara (H9). Of the nine participating hospitals, seven are general hospitals; the others are specialized in infectious diseases (H1) and gastroenterology (H5), respectively. The study data were collected, anonymized, and provided by the National Institute for Public Health.

### Definitions

**Diagnosis**

A confirmed CDI was defined according to the criteria of the European Society of Clinical Microbiology and Infectious Diseases (7). As the sensitivity of toxin detection assays is variable, some cases were considered as “probable CDI” to avoid false negative results. Probable CDI included cases presenting with consistent risk factors and clinical manifestations, negative microbiological tests for CDI and for other etiologies, and a physician’s recommendation for treatment of CDI.

***The origin of Clostridium difficile infections***

The origin of CDI as healthcare-associated (HA) CDI, community-acquired (CA) CDI, or indeterminate was established according to the criteria previously described by Cohen et al. (8).

***Clinical and epidemiological data collection***

The following data were recorded for each patient: demographics, origin and outcome of CDI, and detection of CDI toxins and ribotype. Hospital prevalence rates were calculated per 10.000 patient-days.

***Microbiological testing***

CDI was diagnosed by detecting toxin A/B in fecal samples obtained from seven of the nine participating hospitals and by polymerase chain reaction (PCR) and/or toxin A/B detection in the other two. Stool samples were collected from the first 20 consecutive patients diagnosed with CDI and sent to the Cantacuzino National Institute for Research (NIR) for ribotyping and to the National Institute for Infectious Diseases (NIID) for real-time PCR identification and moxifloxacin susceptibility testing of *C. difficile*.

***Toxigenic culture of Clostridium difficile***

At the NIID and NIR, fecal samples were inoculated on the commercial medium CLO (BioMerieux, France) and a *C. difficile* selective agar and then incubated at 37 °C in an anaerobic atmosphere. The presumptive *C. difficile* colonies were subcultured on Columbia agar medium supplemented with 5% sheep blood (BioMerieux, France). *C. difficile* was identified with a Vitek 2 Compact System (BioMerieux, USA) followed by detection of toxins A/B with a *C. difficile* Test Kit (Biotec, Spain).

***Antimicrobial susceptibility testing***

The identified toxigenic strains were then tested at the NIID for moxifloxacin susceptibility using E-test strips (BioMerieux, USA). The European Committee on Antimicrobial Susceptibility Testing 2014 epidemiological cutoff of 4 µg/mL was used. Due to financial limitations, only the first 80 isolates were tested for moxifloxacin resistance.

***The detection of the genes encoding Clostridium difficile toxins***

Real-time PCR was used for detecting the C. difficile gene encoding toxin B directly from stool samples using the Xpert *C. difficile* assay (Cepheid, USA). The test also allows presumptive identification of strains of *C. difficile* 027 by detecting genes encoding binary toxin subunits A and B (*cdt*A, *cdt*B) and deletion 117 in the *tcd*C gene.

***Clostridium difficile polymerase chain reaction ribotyping***

Molecular typing of *C. difficile* isolates was performed at the NIR by PCR ribotyping in agarose gels, as previously described (9). DNA was extracted from *C. difficile* strains using a Chelex resin kit (InstaGene matrix, Bio-Rad) and then used as a template for PCR amplification of 16S and 23S rRNA genes, as previously described (10). The amplification products were visualized on high-resolution 3% MetaPhor agarose gels (Lonza, Rockland, ME, USA) stained with ethidium bromide. The resulting ribotype profiles were identified using a collection of *C. difficile* strains from the European Center for Disease Prevention and Control-Brazier library of reference ribotypes (11,12).

### Statistical analysis

Categorical variables were reported as percentages, and differences between groups were assessed using Fisher’s exact test. All statistical analyses were performed using SPSS 11.0 (SPSS Inc., Chicago, IL, USA). p<0.05 was considered statistically significant.

## RESULTS

### Patients and *Clostridium difficile* infections diagnosis

During the 4 months of the survey, 393 patients were diagnosed with CDI, among which 255 had a confirmed CDI and 138 had a probable CDI. The CDI diagnosis was confirmed at local laboratories by *C. difficile* toxin detection for 126 patients and by PCR detection of *C. difficile* toxin genes for 129 patients. The mean prevalence during the surveillance period was 5.19 CDI cases per 10.000 patient-days. The highest prevalence rates of CDI were observed in the two monospeciality hospitals, H5 (24.86 per 10.000 patient-days) and H1 (20.02 per 10.000 patient-days). At other hospitals, the prevalence rates ranged from 1.37 in H4 to 14 per 10.000 patient-days in H2 (Table 1). The proportion of patients aged >65 years was 56% (220 cases), and the median age was 67 years (range: 2-94 years). Of the 393 CDI cases, 277 (70.5%) were HA-CDI, 40 (10.2%) were CA-CDI, and the remaining 76 (19.3%) were of indeterminate origin. Overall, HA-CDI cases were predominant in each hospital, but the HA-CDI/CA-CDI ratio ranged from 2.2 at H7 to 17.2 at H6. All but one of the 13 tested isolates from CA-CDI cases belonged to the PCR-027 ribotype and/or were binary toxin positive.

### Molecular characterization of *Clostridium difficile*

PCR for binary toxin gene detection was positive in 150 stool samples. PCR ribotyping was successful in 87 isolates, resulting in the identification of 11 distinct ribotypes. Ribotype 027 was present in 72 of the 87 ribotype-characterized isolates, representing 82.6% (95% CI: 73.6%-89%). The other ribotypes were 002 (four cases); 018 and 087 (three isolates each); 014 (two cases); and 001, 011, 012, 017, 020, and 106 (one isolate each). Overall, 118 of the 150 positive stool samples had the binary toxin gene, representing 78.7% (95% CI: 71.4%-84.5%). These isolates were predominant at each of the participating hospitals ranging from 69.2% to 100% (Table 2).

### *Clostridium difficile* infections outcomes

Forty-five patients (12.3%) with a reported outcome developed severe disease. Of those, 32 patients had a fatal outcome (crude mortality rate = 8.8%), seven patients (1.9%) recovered after intensive care, and the remaining six patients (1.6 %) survived after colectomy. The proportion of severe cases ranged between 5.9% at H8 and 25% at H2. The proportions of severe CDIs caused by ribotype 027 (15.4%) and non-027 ribotypes (18.5%) were similar (p=0.80).

### Moxifloxacin resistance of *Clostridium difficile* isolates

The first 80 *C. difficile* isolates were tested for moxifloxacin susceptibility. The minimum inhibitory concentration (MIC) of moxifloxacin for 59 isolates (73.8%) was >4 μg/mL; 54 of the 59 isolates were highly resistant to moxifloxacin (MIC ≥32 μg/mL). The proportion of moxifloxacin-resistant isolates was significantly higher in isolates identified as or presumed to be ribotype 027 (85.5%) than in other ribotypes (33.3%) (p<0.0001), and 79.5% of the isolates with very high moxifloxacin resistance were 79.5% positive versus 27.8% with other ribotypes (Z score = 4.088, p<0.0001).

## DISCUSSION

The epidemiology of CDI has been predominantly investigated in developed countries; however, few data are available in less developed countries and are reported mainly via case reports or reports of isolated hospital outbreaks (13,14,15). The incidence of ribotype 027 may be underevaluated in countries with limited resources owing to its recent emergence and/or limited availability of diagnostic testing. In the present survey, the estimated prevalence of CDI was lower than the European mean prevalence of seven cases/10.000 patient-days reported by the Euclid study (16). The case density was higher in specialized hospitals (H1 and H5) where patients with diarrhea are usually admitted. Recurrent CDI cases were exclusively managed at these hospitals. However, our data indicated a significant circulation of ribotype 027 in Romania. The global incidence of CDI may be underevaluated. Recently, a point prevalence study conducted at 482 hospitals across 20 European countries reported a high rate (23%) of false negative diagnosis, owing to the limitations of the toxin detection assays (16). Overall, the proportion of missed cases may be higher in areas with both a low sensitivity of toxin detection and a limited awareness of CDI among physicians involved in the management of patients with diarrhea (17). The predominance of ribotype 027 has been repeatedly observed since 2011 in patients with CDI admitted to hospitals in Bucharest, the largest Romanian city (6,18), but there is a lack of ribotype prevalence data from other areas of the country. Our results demonstrated that ribotype 027 is most probably predominant at the national level because >75% of the tested *C. difficile* strains belonged to ribotype 027 and/or were binary toxin positive. There were no significant differences in the prevalence rates of ribotype 027 in any of the participating hospitals. Moreover, the emergence of this epidemic strain is consistent with the other clinical and epidemiological characteristics of CDI reported in this survey, i.e., nearly half the patients were aged <65 years, community-acquired CDIs were reported, the prevalence rate of severe CDI cases and mortality were non-negligible. A study by Spina et al. (19) found that the prevalence of CDI among patients with severe community-acquired diarrhea was higher in Romania than in other European countries. In the present study, nearly 80% of the ribotype 027 isolates were moxifloxacin resistant, which is consistent with the recently reported emergence of moxifloxacin resistance in ribotype 027 and several other ribotypes (20). Our study has several limitations. First, the prevalence of CDI in the participating hospitals may have been underestimated due to the use of several toxin detection tests with variable sensitivity. Second, not all participating hospitals collected samples from the planned number of patients, which is why we included more than 20 patients from two participating hospitals. Third, our study was performed at tertiary-level hospitals, where patients may have a more severe illness than patients admitted to smaller hospitals. Consequently, the severity of CDI described in this study may be overestimated compared with that reported in nationwide data. Fourth, a probable diagnosis was frequent at all participating hospitals and may have an impact on the final results. However, the proportions of probable CDI in both HA-CDI and CA-CDI were similar, suggesting that the CDI analysis was not biased by the inclusion of patients with other etiologies among those with probable CA-CDI. Despite these limitations, the present survey provides important epidemiological data of CDI in Romania that confirms a clinically significant circulation of a highly moxifloxacin-resistant ribotype 027 at a national level. Therefore, preventive strategies including increased awareness should be considered to decrease the mortality and morbidity caused by CDI and healthcare costs associated with CDI. A National Surveillance System of CDI was launched in Romania in August 2014, a few months after completing the analysis of the data collected in this survey.

## Figures and Tables

**Table 1:**
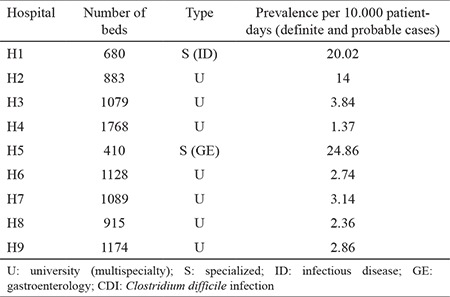
Collaborating hospitals and prevalence of *Clostridium difficile* infection during the study period

**Table 2:**
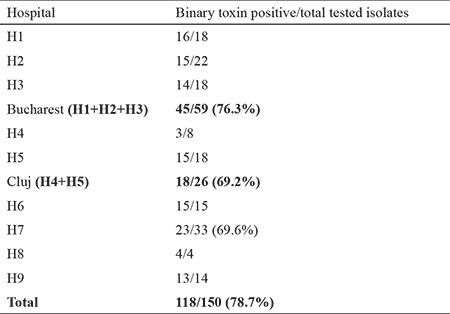
Binary toxin positive *Clostridium difficile* isolates in the participating hospitals

## References

[1] Dallal RM, Harbrecht BG, Boujoukas AJ, Sirio CA, Farkas LM, Lee KK, et al (2002). Fulminant Clostridium difficile: an underappreciated and increasing cause of death and complications. Ann Surg.

[2] Eggertson L (2005). Quebec puts up 20 million dollars for C. difficile fight. C Med Assoc J.

[3] Smith A (2005). Outbreak of Clostridium difficile infection in an English hospital linked to hypertoxin-producing strains in Canada and the US. Eur Surveill.

[4] Kuijper EJ, Coignard B, Brazier JS, Suetens C, Drudy D, Wiuff C, et al (2007). Update of Clostridium difficile-associated disease due to PCR ribotype 027 in Europe. Euro Surveill.

[5] Wilcox MH, Shetty N, Fawley WN, Shemko M, Coen P, Birtles A, et al (2012). Changing epidemiology of Clostridium difficile infection following the introduction of a national ribotyping-based surveillance scheme in England. Clin Infect Dis.

[6] Rafila A, Indra A, Popescu GA, Wewalka G, Allerberger F, Benea S, et al (2014). Occurrence of Clostridium difficile infections due to PCR ribotype 027 in Bucharest, Romania. J Infect Dev Ctries.

[7] Debast SB, Bauer MP, Kuijper EJ; European Society of Clinical Microbiology and Infectious Diseases (2014). European Society of Clinical Microbiology and Infectious Diseases: update of the treatment guidance document for Clostridium difficile infection. Clin Microbiol Infect.

[8] Cohen SH, Gerding DN, Johnson S, Kelly CP, Loo VG, McDonald LC, et al (2010). Clinical practice guidelines for Clostridium difficile infection in adults: 2010 update by the society for healthcare epidemiology of America (SHEA) and the infectious diseases society of America (IDSA). Infect Control Hosp Epidemiol.

[9] Bidet P, Barbut F, Lalande V, Burghoffer B, Petit JC (1999). Development of a new PCR-ribotyping method for Clostridium difficile based on ribosomal RNA gene sequencing. FEMS Microbiol Lett.

[10] O’Neill GL, Ogunsola FT, Brazier JS, Duerden BI (1996). Modiﬁcation of a PCR-ribotyping method for application as a routine typing scheme for Clostridium difﬁcile. Anaerobe.

[11] European Clostridium difficile infection study network (ECDISNET). Europe: Supporting capacity building for surveillance of Clostridium difficile..

[12] Knetsch CW, Lawley TD, Hensgens MP, Corver J, Wilcox MW, Kuijper EJ (2013). Current application and future perspectives of molecular typing methods to study Clostridium difficile infections. Euro Surveill.

[13] Cheng JW, Xiao M, Kudinha T, Xu ZP, Hou X, Sun LY, et al (2016). The First Two Clostridium difficile Ribotype 027/ST1 Isolates Identified in Beijing, China-an Emerging Problem or a Neglected Threat?. Sci Rep.

[14] Balassiano IT, Yates EA, Domingues RM, Ferreira EO (2012). Clostridium difficile: a problem of concern in developed countries and still a mystery in Latin America. J Med Microbiol.

[15] López-Ureña D, Quesada-Gómez C, Miranda E, Fonseca M, Rodríguez-Cavallini E (2014). Spread of epidemic Clostridium difficile NAP1/027 in Latin America: case reports in Panama. J Med Microbiol.

[16] Davies KA, Longshaw CM, Davis GL, Bouza E, Barbut F, Barna Z, et al (2014). Underdiagnosis of Clostridium difficile across Europe: the European, multicentre, prospective, biannual, point-prevalence study of Clostridium difficile infection in hospitalised patients with diarrhoea (EUCLID). Lancet Infect Dis.

[17] Barbut F, Ramé L, Petit A, Suzon L, de Chevigny A, Eckert C, et al (2015). Prevalence of Clostridium difficile infection in hospitalized patients with diarrhea: results of a French prospective multicenter bi-annual point prevalence study. Presse Med.

[18] Benea S, Popescu GA, Badicut I, Florea D, Petrache D, Gavriliu L, et al (2012). Clostridium difficile infections hospitalized in Romanian Institute of Infectious Diseases during the first three months of 2012. 4th International C. difficile Symposium.

[19] Spina A, Kerr KG, Cormican M, Barbut F, Eigentler A, Zerva L, et al (2015). Spectrum of enteropathogens detected by the FilmArray GI Panel in a multicentre study of community-acquired gastroenteritis. Clin Microbiol Infect.

[20] Barbut F, Mastrantonio P, Delmée M, Brazier J, Kuijper E, Poxton I, et al (2007). Prospective study of Clostridium difficile infections in Europe with phenotypic and genotypic characterisation of the isolates. Clin Microbiol Infect.

